# Are Psychology Journals Anti-replication? A Snapshot of Editorial Practices

**DOI:** 10.3389/fpsyg.2017.00523

**Published:** 2017-04-11

**Authors:** G. N. Martin, Richard M. Clarke

**Affiliations:** ^1^School of Psychotherapy and Psychology, Faculty of Humanities, Arts and Social Sciences, Regent’s University LondonLondon, UK; ^2^Department of Infectious Disease Epidemiology, Faculty of Epidemiology and Population Health, London School of Hygiene and Tropical MedicineLondon, UK

**Keywords:** replication, psychology, p-hacking, journal editorial practices, publication bias

## Abstract

Recent research in psychology has highlighted a number of replication problems in the discipline, with publication bias – the preference for publishing original and positive results, and a resistance to publishing negative results and replications- identified as one reason for replication failure. However, little empirical research exists to demonstrate that journals explicitly refuse to publish replications. We reviewed the instructions to authors and the published aims of 1151 psychology journals and examined whether they indicated that replications were permitted and accepted. We also examined whether journal practices differed across branches of the discipline, and whether editorial practices differed between low and high impact journals. Thirty three journals (3%) stated in their aims or instructions to authors that they accepted replications. There was no difference between high and low impact journals. The implications of these findings for psychology are discussed.

## Introduction

The recent ability, or inability, of psychology to replicate novel or well-known, classic findings in the discipline has led to the controversial, but by no means generally accepted, conclusion that psychology is undergoing a “replication crisis” (Pashler and Harris, 2012; Pashler and Wagenmakers, 2012; Laws, 2013; American Psychological Society, 2015; Earp and Trafimow, 2015; Maxwell et al., 2015). Reproducibility of results and the replication of findings is crucial for the development of any science as it transforms a single item of information into a coherent body of demonstrable knowledge and can establish that a reported finding is reliable, robust and consistently obtained. As Simons (2013, p. 79) notes “direct replication by other scientists is the only way to verify the reliability of an effect.” In psychology, the reported crisis seems to be twofold: (1) the discipline has bemoaned a historical failure to publish negative results (which may arise from failed replications), and a preference for the publication of positive results, the so-called publication bias, and (2) when these replications occur, they are unlikely to support the original studies. In short, the discipline has not published enough replications and, when it does, the replications are negative.

Klein et al. (2014), for example, reporting the first of the Many Labs projects hosted by the Open Science Foundation, found a reasonably good rate of replication attempts: Of 13 replication attempts of classic and contemporary findings in social and cognitive psychology using 36 samples comprising 6344 participants, 10 were successful, 1 was weakly replicated and 2 sets of findings were not. A successful replication was one that is considered to produce the same (or greater) effect in the replication as in the original. Both failures to replicate in this study involved social priming. The latest set of replication attempts by the Open Science Collaboration (2015) found that of 100 experiments in cognitive and social psychology published in the journals, *Journal of Experimental Psychology: Learning, Memory and Cognition* (*N* = 28), *Psychological Science* (*N* = 40), and *Journal of Personality and Social Psychology* (*N* = 22) in the year 2008, only 36% were successfully replicated, compared to the positive findings reported in 97% of the original studies.

The studies reported in these papers were largely direct replications. Direct replications are those which faithfully reproduce the methods and materials used in the original study and ensure that the repeated experiment follows as closely as possible the procedure, details and demands of the original research (Nosek et al., 2012). They should minimize the effect of ‘moderator variables,’ variables which may have been present in the replication that were not present in the original report and which are often cited by the authors of studies whose findings were not replicated as the reason for the failure to replicate. Conceptual replications are more fluid. These repeat the original experiment but might alter the procedure or materials or participant pool or independent variable in some way in order to test the strength of the original research, and the reliability and generalisability of the original result. The argument follows that if an effect is found in situation X under condition Y, then it should also be observed in situation A under condition B.

The replication failure is not limited to psychology. Only 11% of 53 landmark preclinical cancer trials were found to replicate (Begley and Ellis, 2012) with 35% of pharmacology studies replicating (Prinz et al., 2011). Of the 49 most widely cited papers in clinical research, only 44% were replicated (Ioannidis, 2005). Sixty per cent failed to replicate in finance (Hubbard and Vetter, 1991), 40% in advertising (Reid et al., 1981), and 54% in accounting, management, finance, economics, and marketing (Hubbard and Vetter, 1996). The situation is better in education (Makel and Plucker, 2014), human factors (Jones et al., 2010), and forecasting (Evanschitzky and Armstrong, 2010).

One ostensible reason for the current turmoil in the discipline has been psychology’s publication bias (Pashler and Wagenmakers, 2012). Publication bias, it is argued, has partly led to the current batch of failed replications because journals have historically been reluctant to publish, or authors have been reluctant to submit for publication, negative results. Single dramatic effects, therefore, may have been perpetuated and supported by the publication of positive studies while negative, unsupportive studies remained either unpublished or unsubmitted. Journals are more likely to publish studies that find statistically significant results (Schooler, 2011; Simmons et al., 2011) and null findings are less likely to be submitted or accepted for publication as a result (Rosenthal, 1979; Franco et al., 2014).

While publication bias is thought to be well-established, there has been no objective method of confirming whether papers have been rejected for reporting negative results, largely because these data are not publicly available. Although Fanelli (2011) found that the proportion of positive results published in peer-reviewed journals increased by 22% between 1990 and 2007, it does not follow that a similar, if any, number of negative results was also submitted and rejected. Fanelli (2010) noted that 91.5% of psychology studies reported data supporting the experimental hypothesis, five times higher than that reported for rocket science. Sterling et al.’s (1995, p. 108) study of 11 major journals concluded that these outlets continued to publish positive results and that the “practice leading to publication bias has not changed over a period of 30 years.” Smart (1964) found that of the five psychology journals examined, the largest percentage of non-significant results published was 17%; the lowest % of positive results was 57%. Coursol and Wagner (1986)’s self-report survey of 1000 members of the APA’s Counseling and Psychotherapy Divisions found that 66% of the authors’ studies reporting positive results were published but only 22% of those reporting neutral or negative results were. While this may indicate a publication bias, the finding may be explained by an alternative reason: the negative studies may have been of poorer quality.

Some journals appear to be reluctant to publish replications (whether positive or negative), and prize originality and novelty. Makel et al. (2012) study of 100 journals with the highest 5-year impact factor found that 1% of published studies were replications. A survey of 429 journal editors and editorial advisors of 19 journals in management and related social sciences, found that the percentage of papers rejected because they were replications ranged from 27 to 69% (Kerr et al., 1977). A study of 79 past and present editors of social and behavioral science journals by Neuliep and Crandall (1990) found evidence of a reluctance to publish negative results, a finding also replicated in Neuliep and Crandall’s (1993a) study of social science journal reviewers- 54% preferred to publish studies with new findings. Nueliep and Crandall’s data suggest that journals will only publish papers that report “original” (sometimes “novel”) data, findings or studies, rather than repeat experiments. Sterling (1959) found in his review of 362 psychology articles that none were replications; Bozarth and Roberts (1972) similarly found that of 1046 clinical/counseling psychology papers published between 1967 and 1970, 1% were replications. However, in their analysis of the type of papers published in the first three issues of the *Journal of Personality and Social Psychology* from 1993, Neuliep and Crandall (1993b) reported that 33 out of 42 of them were conceptual or direct replications.

As with replication failure, failure to publish replications is not unique to psychology- 2% of 649 marketing journal papers published in journals between 1971 and 1975 (Brown and Coney, 1976), and 6% of 501 advertising/communication journal papers published between 1977 and 1979 (Reid et al., 1981) were replications. Hubbard and Armstrong (1994) found that none of the 835 marketing papers they reviewed were direct replications. In 18 business journals, 6.3% of papers published between 1970 and 1979 and 6.2% of those published between 1980 and 1991 were replications. In 100 education journals, 461 (of 164, 589) papers were replications and only 0.13% of these were direct replications (Makel and Plucker, 2014), eight times smaller than the percentage seen in psychology journals (Makel et al., 2012). In forecasting research, 8.4% of papers published in two journals between 1996 and 2008 were replications (Evanschitzky and Armstrong, 2010); in marketing, 41 of 2409 (1.17%) of papers in five journals published between 1990 and 2004 were replications (Evanschitzky et al., 2007).

There is some indirect evidence, therefore, to suggest that editors and reviewers are reluctant to accept replications. However, the evidence is primarily anecdotal, based largely on data provided by surveys of selected editorial staff and reviewers’ views, and on *post hoc* examination of journal output where the processes leading to the output decisions are unknown. In order to provide an objective analysis of journal and editors’ explicit guidance to authors regarding the value and acceptance of replication studies, we examined psychology and psychology-related journals’ instructions to authors and journal aims and scope to determine whether (i) journals specifically accepted, discouraged or prohibited the submission of replications, (ii) acceptance of replications differed by branch of the discipline, (iii) whether journals with a high impact factor differed from those with a low impact factor.

## Materials and Methods

The “instructions to authors” and “aims and scope” of peer-reviewed journals publishing psychological research were reviewed in the Summer of 2015. A list of psychology and psychology-related journals was obtained first through the selection of common publishers of psychology journals (Sage, Taylor and Francis, Elsevier, Springer, Wiley). From these, all journals within the subheading of Psychology, or using the search criteria “Psychology,” were selected. This sample was then cross referenced with freely available online lists of the top 100 psychology journals ranked by the journal’s eigenfactor and impact factor so as to obtain top journals independently published by APA, Cambridge or others. From this initially created list of journals, all non-English language and multiple entries were removed. Each journal on the list was then visited online its aims and scope section was reviewed along with any additional information that pertained to the content of articles accepted.

One thousand, one hundred and fifty one journals were identified as psychology journals (e.g., *Journal of Experimental Psychology: General, Frontiers in Psychology*) or psychology-related journals (e.g., *British Journal of Learning Disabilities, Journal of Employment Counseling, American Journal of Family Therapy*). The number of journals whose editorial guidelines specifically stated the acceptance of replications was calculated and compared with those which did not. The number of journals accepting replications was also calculated according to the journal’s impact factor (stated on the journal’s website and cross-checked through online databases) and a comparison was made between those journals rated above and below the mean impact factor. Finally, the number of journals accepting replications was calculated according to the branch of the discipline they primarily published in (e.g., general, cognitive psychology, social psychology), as seen in **Table 1**.

**Table 1:** Acceptance of replications in psychology journals by branch of the discipline.

Branch	Number of journals accepting replications (Total number of journals, in brackets)	Number of journals with an IF > mean IF which accept replications (Total number of journals with IF > mean, in brackets)	Number of journals with an IF < mean which accept replications (Total number of journals with IF < mean, in brackets)	Number of journals not publicly reporting an IF and which accept replications (Total number of journals not publicly reporting an IF, in brackets)
General	4 (103)	2 (30)	2 (47)	0 (26)
Cognitive	5 (123)	2 (39)	3 (64)	0 (20)
Social	4 (93)	1 (14)	2 (56)	1 (23)
Clinical	0 (78)	0 (25)	0 (35)	0 (18)
Developmental	2 (130)	0 (36)	1 (61)	1 (33)
Sports	1 (12)	0 (2)	1 (8)	0 (2)
Health	1 (110)	0 (33)	0 (41)	1 (36)
Educational	3 (85)	1 (5)	2 (42)	0 (38)
Occupational	1 (67)	0 (17)	0 (35)	1 (15)
Personality/Individual differences	4 (37)	3 (16)	1 (19)	0 (2)
Counseling/Psychotherapy	2 (126)	2 (10)	0 (35)	0 (81)
Behaviorism	1 (14)	0 (0)	0 (8)	1 (6)
Cross-cultural/Minority psychology	2 (46)	0 (0)	2 (12)	0 (34)
Forensic	0 (53)	0 (9)	0 (27)	0 (17)
Biological/Neuropsychology	3 (67)	0 (31)	3 (24)	0 (12)
Evolutionary psychology	0 (7)	0 (2)	0 (1)	0 (4)
**Total**	33 (1151)	11 (269)	17 (515)	5 (367)

## Results

Of the 1151 journals reviewed, 33 specifically stated that replications would be accepted- approximately, 3%. The mean impact factor for journals with IFs was 1.93. When we examined whether there was difference in journal practices between those with an impact factor above or below this mean, we found no difference in replication acceptance 10 [x^2^ (1,*N* = 784) = 0.55, *p* = 0.46, Cohen’s *d =* 0.0529].

When the journals were examined for the specific wording they used in their aims and instructions, we were able to identify four broad types of publication: (1) Journals which stated that they accepted replications; (2) Journals which did not state they accepted replications but did not discourage replications either; (3) Journals which implicitly discouraged replications through the use of emphasis on the scientific originality of submissions, and (4) Journals which actively discouraged replications by stating explicitly that they did not accept replications for publication.

The percentage of journals in each of these categories were 3% (*N* = 33, category 1); 63% (*N* = 728, category 2); 33% (*N* = 379, category 3); and 1% (*N* = 12, category 4). Of the journals in category 3, 104 indicated in the first line of their aims and scope that they preferred the submission of original research.

We were able to determine the primary branch of psychology for all journals. The number of journals accepting replications, according to branch, is listed in **Table 1**. There were no significant differences between different branches of psychology in terms of their acceptance of replication [x^2^ (15,*N* = 1152) = 21.02, *p* = 0.14, *C* = 0.134]. No journal in clinical, forensic, health or evolutionary psychology explicitly accepted replications.

## Discussion

The aim of this study was to investigate whether peer-reviewed journals publishing research in psychology stated that they accepted submissions in the form of replications. Of the 1151 journals included in this study, 33 explicitly stated that replications were accepted or encouraged. There were no differences between branches of the discipline in terms of the numbers of journals accepting replications nor between journals with high or low impact factors.

It is clear that the vast majority of journals in psychology rarely ever encourage the submission of replications: only 3% do. A typical statement is that provided by the *International Journal of Behavioral Development*, for example: “*Studies whose sole purpose is to replicate well-established developmental phenomena in different countries or (sub) cultures are not typically published in the International Journal of Behavioral Development.”* This prescription is not unique to this journal.

The findings are consistent with other studies which have provided partial, anecdotal evidence that editorial practices in other journals tend to discourage the submission of replications (e.g., Kerr et al., 1977; Neuliep and Crandall, 1990, 1993a,b; Makel et al., 2012). The findings from this study extend previous work by demonstrating that journals’ stated editorial practices do not explicitly encourage the submission of replications. Of course, it is possible that such editorial practices actually reflect publishers’ practices and wishes, rather than those of the editors. Academic publishing is a commercial enterprise, as well as an academic one, and publishers may wish to see published in their journals findings that are unique and meaningful and which will increase the journals’ attractiveness, submission rates and, therefore, sales. No study has examined publishers’ views of replication and this might be an avenue of research for others to consider.

Our data also reflect the aims and scope of journal editorial policies which explicitly mention replications and encourage the submission of papers which constitute replications. The conclusion cannot be drawn that 1, 218 journals do not or would not accept replications. However, it is noteworthy that, despite the establishment of the Open Science Framework in 2011, the number of special issues of journals in psychology dedicated to replications in recent years, and the controversy over psychology’s “replication crisis,” that so few journals in Psychology explicitly encourage the submission of replications and that 379 journals emphasize the originality of the research in their aims and scope. If it is agreed that that science proceeds by self-correction and that it can only progress by demonstrating that its effects and phenomena are valid and can be reliably produced, it is surprising that so few journals appear to reflect or embody this agreement explicitly in their editorial practices. An analysis of other disciplines’ practices is underway to determine whether the data are reflective only of editorial practices in psychology or are common in other sciences. It is also worth acknowledging that this study was conducted in the Summer of 2015. A repeat survey of the same journals (and any new journals) in 2017 might be informative.

The issue of failing to accept replications is also tied to the issue of publication bias- the publication of positive results and the discouragement of the publication of negative results: arguably, the Scylla and Charybdis of psychological science in the early 21st century. As Smart (1964, p. 232) stated, “withholding negative results from publication has a repressive effect on scientific development.”

However, we would suggest that there are reasonably straightforward solutions to this problem and a number of specific solutions were suggested by Begley and Ellis (2012) in their discussion of replication failure in preclinical cancer trials. For example, Begley and Ellis (2012) recommend that there should be more opportunities to present negative results. It should be an “expectation” that negative results should be presented in conferences and in publications and that “investigators should be required to report all findings regardless of outcome” Begley and Ellis (2012, p. 533), a suggestion which has given rise to the encouragement of pre-registered reports whereby researchers state in advance the full details of their method (including conditions and numbers of participants required) and planned statistical analysis and do not deviate from this plan. They argue that funding agencies must agree that negative results can be just as informative and as valuable as positive results.

Another solution would be for any author who submits a statistically significant empirical study for publication also conducts and submits along with the original study at least one replication, whether this replication is statistically significant or not. Such reports might even be pre-registered. It is arguable that some journals such as *Journal of Personality and Social Psychology* and some from the *JEP* stable have historically published protracted series of studies within the same paper. This is true, although these portfolio of studies either conceptually replicate the results of the main study or develop studies which expand on the results of the original study. Our proposal is that each second study should be a direct replication with a different sample which meets the recruitment criteria of the original study. Of course, this proposal is also open to the valid criticism that an internal (if direct) replication attempt will lead to a positive result because the literature notes that replications are more successful if conducted by the same team as the original study. However, this would be one step toward (i) ensuring that the importance of replication is made salient in the actions and plans of researchers and (ii) providing some sort of reliability check on the original study.

We might also suggest a more radical and straightforward solution. We would recommend that all journals in psychology (1) explicitly state that they accept the submission of replications and (2) explicitly state that they accept replications which report negative results. That is, these two recommendations should be embedded in the aims and the instructions to authors of all psychology journals. If such recommendations were to become the norm and encourage and make common the practice of publishing replicated or negative work, psychology could demonstrably put its house in order. Where psychology leads, other disciplines could follow.

## Author Contributions

GNM devised the original idea and method; RC conducted the research and completed the analysis; GNM wrote the first draft of the paper; RC revised this draft; both authors contributed to revisions. Both consider themselves full authors of the paper, and the order of authorship reflects the input provided.

## Conflict of Interest Statement

The authors declare that the research was conducted in the absence of any commercial or financial relationships that could be construed as a potential conflict of interest.
